# Molecular characterisation of extracellular vesicles released by *Strongyloides stercoralis* infective larvae isolated from a clinical sample

**DOI:** 10.1186/s13071-026-07484-0

**Published:** 2026-06-03

**Authors:** Michela Deiana, Laura Veschetti, Kieran Reynolds, Vicky L. Hunt, Nicole Padovani, Marcello Manfredi, Elisabetta Vezzelli, Eleonora Rizzi, Monica Degani, Giovanni Malerba, Tamara Ursini, Niccolò Ronzoni, Chiara Piubelli, Dora Buonfrate, Natalia Tiberti

**Affiliations:** 1 Department of Infectious, Tropical Diseases and Microbiology, IRCCS Sacro Cuore Don Calabria Hospital, Negrar di Valpolicella, Italy; 2 Infections and Cystic Fibrosis Unit, Division of Immunology, Transplantation and Infectious Diseases, IRCCS San Raffaele Scientific Institute, Milan, Italy; 3 Vita-Salute San Raffaele University, Milan, Italy; 4 Department of Life Sciences, University of Bath, Bath, BA2 7AY UK; 5 Department of Engineering for Innovation Medicine, University of Verona, Verona, Italy; 6 Department of Translational Medicine, University of Piemonte Orientale, Novara, Italy; 7 Institute for Molecular and Translational Cardiology (IMTC), IRCCS Policlinico San Donato, S. Donato Milanese, Milan, Italy; 8 GM Lab, Department of Surgical Sciences, Dentistry, Gynaecology and Paediatrics, University of Verona, Verona, Italy

**Keywords:** *Strongyloides stercoralis*, iL3, Extracellular vesicles, miRNA, smallRNA

## Abstract

**Background:**

Extracellular vesicles (EVs) represent a key mechanism of host–pathogen crosstalk. Numerous helminth parasites have already been reported to shed EV-like structures carrying biomolecules, including small RNAs (sRNAs), with functional effects on target cells. However, the ability of *Strongyloides stercoralis* to release EVs has yet to be demonstrated.

**Methods and results:**

Following the isolation of *S. stercoralis* infective larvae (iL3s) from faecal samples obtained from a patient with strongyloidiasis, we showed that iL3s maintained in vitro for up to 48 h release EV-like structures. Transmission electron microscopy and nanoparticle tracking analysis highlighted vesicular structures enclosed by a bilayer and with a diameter of 120 nm in range. Small RNA sequencing identified multiple EV-associated sRNA types, including miRNAs, only partly overlapping with the previously described somatic miRNome. Comparative analyses revealed that several EV-associated miRNAs were conserved amongst *Strongyloides* spp., whereas others appeared specific to *S. stercoralis*. Prediction analyses indicated that miRNAs and other sRNAs may target human genes associated with the regulation of gene expression and immune response, supporting a potential role in host–parasite interaction.

**Conclusions:**

These findings provide the first experimental evidence that *S. stercoralis* iL3s release EVs carrying regulatory sRNAs and suggest that EV-mediated RNA delivery may represent an additional tool for host–pathogen interaction. More in-depth investigations of these EVs may provide novel insights into the pathophysiology of strongyloidiasis as well as novel targets for clinical applications.

**Graphical Abstract:**

**Figure:**
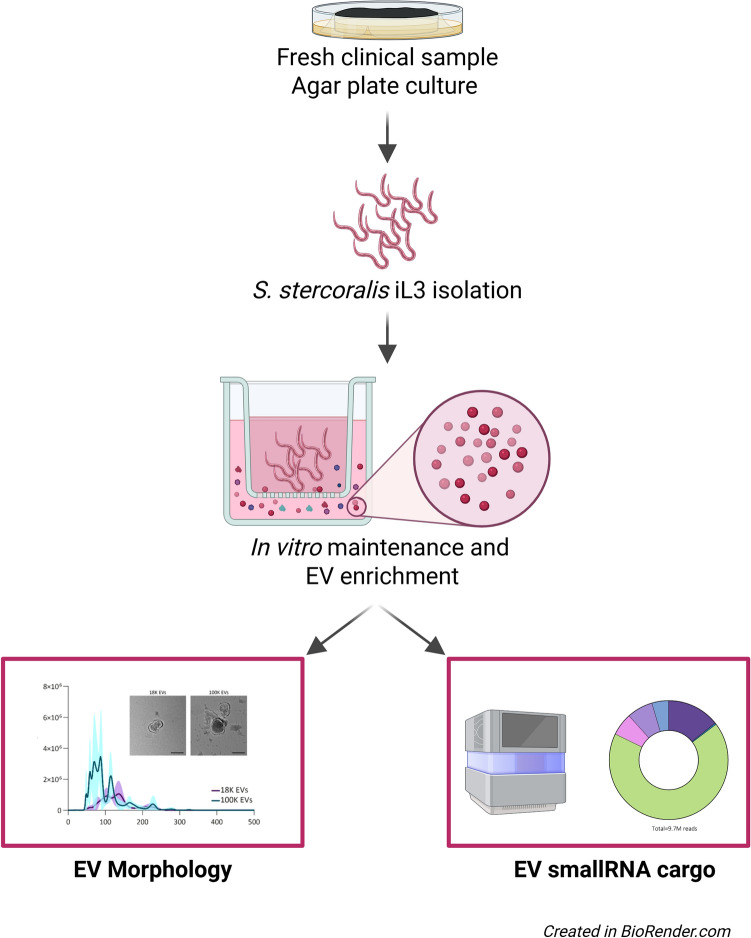

**Supplementary Information:**

The online version contains supplementary material available at 10.1186/s13071-026-07484-0.

## Background

Strongyloidiasis, caused by the soil-transmitted helminth *Strongyloides stercoralis*, affects millions of people mainly in tropical and sub-tropical regions, particularly in resource-limited settings with poor sanitation [1]. Human infection is acquired through contact with soil contaminated with free-living infective larvae (iL3s) and is maintained chronically due to its capacity of autoinfection. Indeed, some L1 rhabditiform larvae can develop into iL3s when still in the host intestine and re-infect the same patient through penetration of the intestinal mucosa or the perianal skin [2, 3]. This unique auto-infective cycle distinguishes *S. stercoralis* from other soil-transmitted helminths and allows the infection to persist even in the absence of re-exposure to an external source of infection. During uncomplicated chronic strongyloidiasis, a balance between autoinfection and the host immune response is essential to prevent uncontrolled worm replication. Disruption to this equilibrium, as in the case of immunosuppression, leads to severe and life-threatening forms of strongyloidiasis (i.e. hyper-infection and dissemination) triggered by an acceleration of the auto-infective cycle [4–6]. Helminths have, in fact, evolved intricate mechanisms of interaction with their hosts to survive relatively adverse conditions. They manipulate the host immune system so that their presence is tolerated without creating excessive damage [7]. Helminth-derived excretory/secretory products (ESP) have been proposed to participate in this interplay with the host immune system. Indeed, ESP were reported as involved in host–parasite crosstalk by promoting immune evasion, pathogen survival and immunomodulation [8–10]. An important component of ESP is represented by extracellular vesicles (EVs) – submicron elements enclosed in a lipid bilayer able to deliver biological material from a parent to a recipient cell, thus modulating gene expression and cellular functions of the latter. In recent years, EVs have emerged as key mediators also in host–helminth interactions. First described in *Echinostoma caproni* and *Fasciola hepatica* [11], helminth-derived EVs – or exosome-like structures – have since been reported to be released by a large number of nematodes, trematodes and cestodes, as summarised elsewhere [12–14]. Over the past decade, the molecular composition, mainly at the protein and smallRNA (sRNA) levels, of EVs derived from various helminths has been characterised and their potential immuno-modulatory role has been explored [11, 13–18]. Hence, EVs are now considered important tools for parasites to interact with the host and influence the progress and maintenance of the infection [19]. In the context of strongyloidiasis, miRNAs and other sRNAs have been identified as expressed by *S. ratti, S. venezuelensis, S. papillosus* and *S. stercoralis*, and predicted to target host genes [20–23].

Despite the expanding body of evidence showing that helminths exploit EVs as specialised tools for communication and immuno-modulation [17, 24], no study has yet demonstrated the release of EVs by *S. stercoralis*. ESP have been investigated in *S. ratti* and *S. venezuelensis*, however, only indirect evaluation of the possible vesicular component was hypothesised [25, 26]. For instance, Soblik and colleagues explored the proteome of *S. ratti* ESP at different developmental stages [27], whilst Roldán-Gonzáles showed that *S. venezuelensis* iL3 ESP were recognised by patients’ serum, and an important portion of the recognised proteins were predicted as released within EVs [26]. To try to contribute to fill this gap of knowledge, here, we provide the first experimental evidence of EVs release by *S. stercoralis* iL3 clinical isolates and used next generation sequencing their cargo of small RNAs, opening novel avenues on the potential mechanisms of parasite communication, immune modulation and chronic infection in human strongyloidiasis.

## Methods

### *Strongyloides stercoralis* iL3 collection, maintenance and EV enrichment

Faeces were obtained from a subject admitted at the IRCCS Sacro Cuore Don Calabria Hospital. Fresh faeces were mixed with charcoal and saline, and cultured on agar plates as reported elsewhere [28]. *S. stercoralis* free-living iL3 were harvested starting from day 3 of culture [29], washed with phosphate buffered saline (PBS) and axenised in PBS supplemented with 100 U/ml penicillin, 100 µg/ml streptomycin and 0.625 µg/ml amphotericin B (all from Gibco, Thermo Fisher Scientific) for 2 h at 4 °C. Larvae were maintained at 20,000 iL3/ml density in RPMI without phenol red (Gibco, Thermo Fisher Scientific), supplemented with 100 U/ml penicillin, 100 µg/ml streptomycin and 0.625 µg/ml amphotericin B in trans-well inserts with 1 μm pore size for 48 h. Microscopic inspection confirmed highly motile larvae after 48 h of in vitro maintenance, although the larval mortality rate was not quantified. Conditioned medium was collected from the lower compartment and replaced with fresh medium after 24 h of maintenance. After collection, the conditioned medium was centrifuged for 5 min at 500 x*g* and the supernatant was further spun at 1800 x*g* for 10 min, aliquoted and stored at −80 °C until further use. EVs were enriched from 5.4 ml of conditioned medium by differential ultracentrifugation, pooling medium collected after 24 and 48 h of in vitro maintenance. Briefly, medium was first centrifuged at 18,000 x*g* for 45 min at 4 °C, and the pellet – containing larger vesicles – was re-suspended in 0.22 μm filtered PBS and washed at 18,000 x*g* for 45 min at 4 °C to obtain 18K EVs. The 18K supernatant was ultra-centrifuged at 100,000 x*g* for 70 min at 4 °C using a Fiberlite F50L-24 × 1.5 Fixed-Angle Rotor on a Sorvall™ WX ultracentrifuge (Thermo Scientific). The 100K pellet (hereafter referred to as 100K EVs) was washed in 0.22 μm filtered PBS at 100,000 x*g* for 70 min at 4 °C.

### EV measurement by nanoparticle tracking analysis and visualisation by transmission electron microscopy (TEM)

Particle size and concentration were analysed by nanoparticle tracking analysis (NTA) using a NanoSight NS300 instrument (Malvern Panalytical, Malvern) equipped with a 488 nm laser and a scientific CMOS camera. EV preparations were vortexed and diluted (1:33) to obtain a concentration and particles/frame within the recommended measurement range. Three consecutive videos of 60 s per sample were acquired with a syringe pump speed set to 20 and subsequently analysed using the NTA 3.4 software (Malvern).

For each EV preparation, 10 μl of sample were adsorbed onto an ultra-thin carbon-coated copper grid (CF200H-Cu-UL, Electon Microscopy Sciences), stained with UranyLess solution (Electron Microscopy Sciences) and air dried before visualisation on a Morgagni 268D (FEI Philips) transmission electron microscope at 80kV.

### Small RNA sequencing and bioinformatics analyses

#### RNA extraction, library preparation and sequencing

Vesicular RNA was enriched from 3.6 ml of conditioned medium using the kit (Qiagen), following manufacturer’s instructions. Briefly, samples diluted with XBP buffer were loaded onto the column for EV binding. After washing, QIAzol reagent was added to lyse and elute the vesicles, followed by phase separation with chloroform. The RNA present in the aqueous phase was collected and purified using the RNeasy MinElute spin column. Vesicular RNA was eluted in 14 µl of RNase-free water.

For smallRNA profiling, 5 µl of RNA were used as input to build the library using the QIAseq miRNA Library Kit (Qiagen). Library quality controls were performed with Qubit™ 4 Fluorometer (Qubit RNA HS Assay Kit, ThermoFisher Scientific) and The 4200 TapeStation System using the High Sensitivity D1000 screen tape (Agilent). Libraries were sequenced on an Illumina NextSeq1000 sequencer (Illumina). Raw reads were quality-checked and aligned to the *S. stercoralis* reference genome (GCA_029582065.1).

#### Analysis of miRNAs

For miRNA prediction and quantification, a catalogue of genomic regions generating miRNA precursors was built. In detail, the quality of raw reads was assessed using FastQC v0.12.1 [30] and Cutadapt v2.8 [31] was used to trim adapter sequences. Only reads with length ≥ 16 bp and ≤ 35 bp, a range compatible with typical miRNA size, were retained for downstream analysis. Reads were aligned to the WormBase ParaSite WBPS19 *S. stercoralis* reference genome (available at: https://ftp.ebi.ac.uk/pub/databases/wormbase/parasite/releases/WBPS19/species/strongyloides_stercoralis/PRJEB528; downloaded on 13 March 2025) using HISAT v2.2.1 [32] to remove potential contaminants. Mapping reads were used as input for miRDeep2 [33] to perform miRNA prediction. Predicted mature miRNAs and their precursors were quantified using the quantifier.pl module of miRDeep2. To annotate the genomic context of regions generating miRNAs, precursor sequences in FASTA format were mapped on the reference genome using National Center for Biotechnology Information (NCBI) Basic Local Alignment Search Tool (BLAST) v2.14.0 + [34]. Matches showing 100% sequence identity and E-value ≤ 0.01 were retained, and the genomic annotation (genic, intergenic or antisense to gene) of each precursor-matching region was extracted using bedtools v2.31.1 [35].

To assess the number of miRNAs shared across eukaryotes, we compared the predicted mature miRNA sequences obtained from EVs against all entries in miRBase v22.1 (available at: https:// www.mirbase.org; retrieved on 27 February 2025). We then focussed on *Strongyloides* spp. and *Caenorhabditis elegans* miRNAs. Briefly, *Strongyloides ratti* sequences present in miRbase were integrated together with the ones from *S. stercoralis* catalogue [23] and *Strongyloides papillosus* miRNAs [22] available in the literature. *C. elegans* miRNAs available in miRbase were also included in the analysis since it is a well-established nematode model organism. The miRNA sequences were considered as shared amongst different organisms when they showed 100% sequence identity. The UpSet plot was generated with the UpSetR package v1.4.0 in an R v4.4.1 environment.

Human 3’-UTR sequences were used together with expressed *S. stercoralis* vesicular mature miRNAs to predict potential human mRNA targets of *S. stercoralis* miRNA. In detail, human 3’-UTR sequences were extracted from the *Homo sapiens* GRCh38.p14 primary assembly genome downloaded from Gencode (available at: https://ftp.ebi.ac.uk/pub/databases/gencode/Gencode_human/release_47; retrieved on 17 October 2024) using the coordinates of the ‘three_prime_UTR’ regions indicated in the primary assembly basic annotation GTF file. Expressed *S. stercoralis* vesicular mature miRNA sequences and human 3’UTR sequences were then used as input for PITA v6 [36] to perform the miRNA target prediction analysis. Results were filtered considering targets having a ddG ≤  − 30.

#### Analysis of other sRNAs

Analysis was performed with HuntLab-smallRNA v2.0.0 (https://github.com/Vicky-Hunt-Lab/HuntLab-smallRNA). In brief, data were pre-processed using the built-in Qiagen kit option, then aligned to the *S. stercoralis* reference genome allowing for one mismatch with Bowtie2 [37]. The pipeline sorted the aligned sRNAs by length and counted frequency by length and first base. Unitas [38] was then run to classify sRNA sequences on the basis of origin sequence. The CDS, mRNA and transposable element sequences were extracted from WormBase ParaSite v19, whilst rRNA and tRNA sequences from RNAcentral [39]. These files were provided as reference files using the refseq parameter. Targets in the human transcriptome were predicted by using Bowtie2 to align reverse complements of the sRNA sequences to the human cDNA sequences downloaded from Ensembl 115 [40], without allowing mismatches. Sequences annotated as miRNA or low complexity were excluded from the analysis. Finally, Gene Ontology (GO) terms and Kyoto Encyclopedia of Genes and Genomes (KEGG) pathway enrichment was analysed using ShinyGO 0.85 with default parameters [41].

## Results

### *Strongyloides stercoralis* infective larvae release EVs

**Fig. 1:**
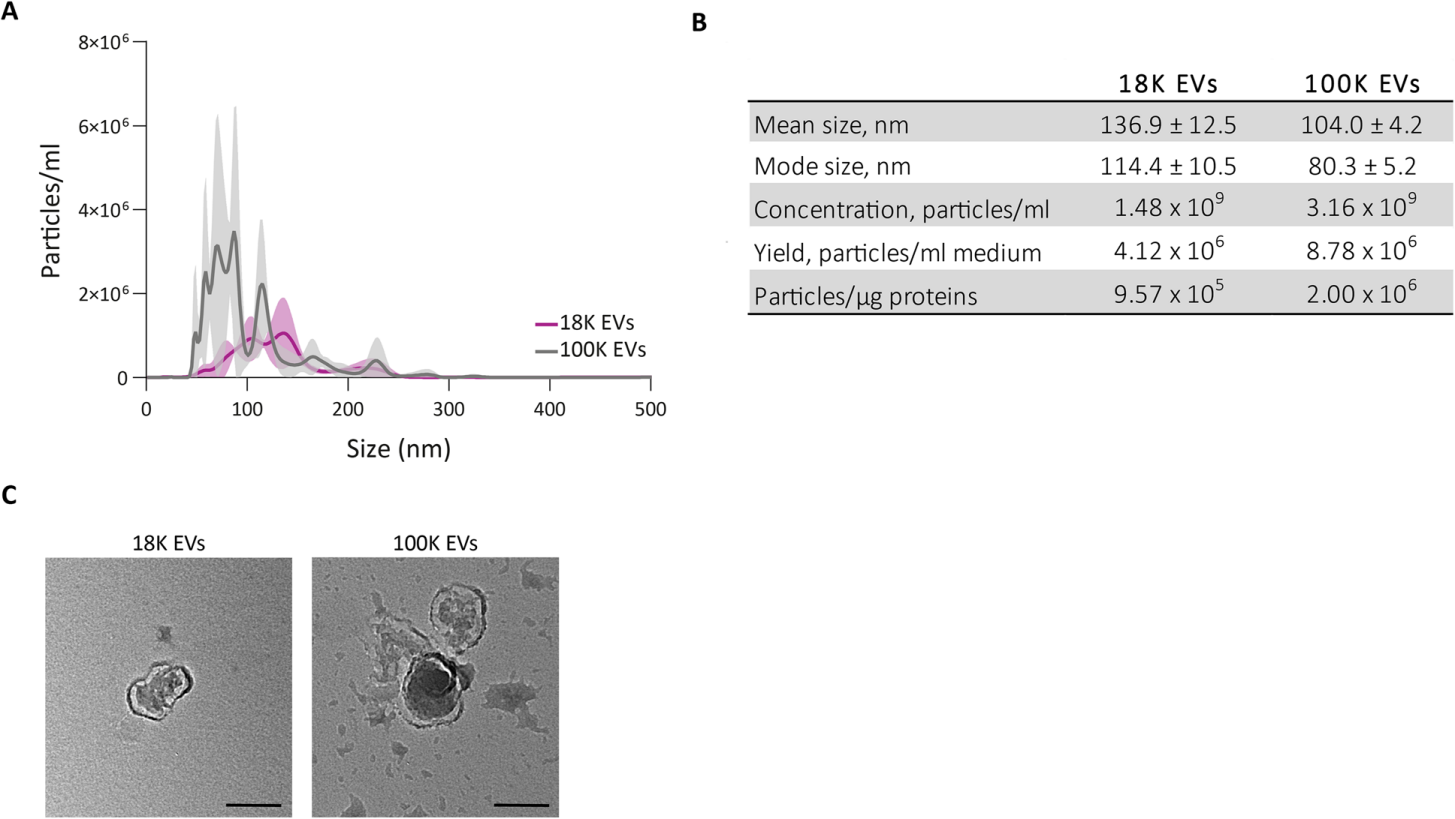
Characterisation of 18K EVs and 100K EVs released by *S. stercoralis* iL3. **A** Size distribution plot of EVs as determined by Nanoparticle Tracking Analysis (NTA). **B** NTA statistics. For each parameter, the mean and standard error (SE) of the three replicate NTA acquisitions are reported. **C** EV visualisation by transmission electron microscopy. Bars = 50 nm. 18K EVs are depicted with a purple line; 100K EVs are depicted with a grey line

Differential ultracentrifugation at two enrichment speeds was employed to assess the presence of EV-like structures in the conditioned medium of *S. stercoralis* iL3s maintained in vitro for up to 48 h. NTA of 18K EVs and 100K EVs revealed the presence of relatively small particles in both preparations. Particle mode diameter was 114.4 nm and 80.3 nm for 18K EVs and 100K EVs, respectively, and particle concentration was two-fold higher in 100K EVs than in 18K EVs (Fig. 1A, B). TEM images further confirmed the presence of vesicular structures in both preparations, revealing particles enclosed by a bi-layered membrane and exhibiting the characteristic cup-shaped morphology of EVs (Fig. 1C).

### *Strongyloides stercoralis* iL3-EVs contain miRNAs that originate from intergenic regions and are predicted to target human genes

Sequencing yielded 14,298,545 reads, 9,748,024 (68%) of which satisfied all quality control requirements, including length between 16 and 35 bp and alignment to the GCA_029582065.1 reference genome (Supplementary Table S1_1). Bioinformatics analysis identified 68 mature miRNAs and precursors (Supplementary Table S1_2). Genomic context analysis detected 319 genomic loci [*n* = 176 on the sense ( +) strand and *n* = 143 on the antisense (–) strand] from which precursor sequences can originate (Supplementary Table S1_3). All originating sites were located in intergenic regions, with 9% forming two clusters: one at positions 471,021–472,073 on SSTP_contig0000007 (*n* = 19) and the other at positions 32,254–35,835 on SSTP_contig0000003 (*n* = 11). The remaining 289 genomic loci did not form clusters. Mature miRNA expression levels were quantified and are reported in Supplementary Table S1_4.

**Fig. 2:**
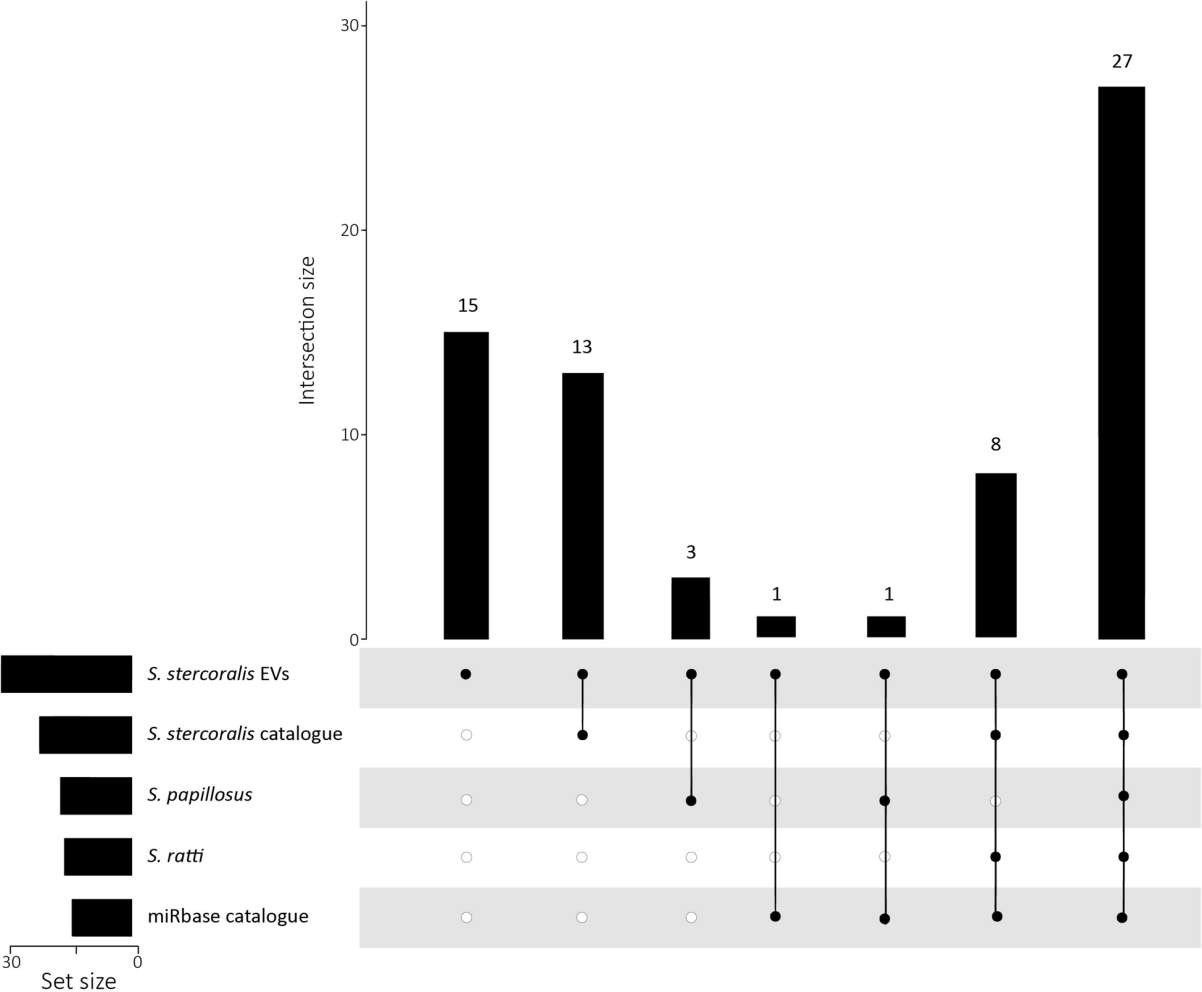
Cross-species comparison of EV-derived mature miRNAs. miRNA sequences shared amongst *S. stercoralis* EVs, *S. stercoralis*, *S. ratti*, *S. papillosus* and miRbase catalogue. Set size indicates the total number of miRNAs identified in each species or dataset, whilst intersection size represents the number of miRNAs with 100% sequence identity with the indicated species

We compared the mature miRNA sequences obtained from *S. stercoralis* iL3-EVs against all entries in miRbase (*n* = 48,885) to explore their conservation across eukaryotic species. Amongst the 68 *S. stercoralis* EVs, 76.5% matched with *Strongyloides* spp. (Supplementary Table S1_5), whilst none with human mature miRNA sequences. A total of 15 sequences (22%) were unique to *S. stercoralis* EVs, whilst 13 (19.1%) were only shared with *S. stercoralis* catalogue [23]. In addition, 39 (57.3%) of the 68 *S. stercoralis* EV miRNAs were shared with *S. papillosus* and/or *S. ratti*, 89.7% of which also overlapped with the published *S. stercoralis* catalogue (Fig. 2).

To investigate potential human targets of *S. stercoralis* EV miRNAs, we predicted their possible interaction with human 3’UTR sequences. Two miRNAs, sts-miR-172-5p and sts-miR-175-5p, were predicted to match seven human genes: *LGR6*, *DZIP3*, *PACSIN1*, *TRA2A, EPHB6, SHC3* and *EMD.* Each miRNA targeted multiple genes, but no gene was targeted by both miRNAs. Notably, sts-miR-175-5p was predicted as present exclusively in *S. stercoralis* EVs. Detailed information on predicted target sites and predicted binding energies is provided in Supplementary Table S1_6.

### *Strongyloides  stercoralis* iL3-EVs contain sRNAs predicted to target human genes involved in the immune response and neuron biology

**Fig. 3:**
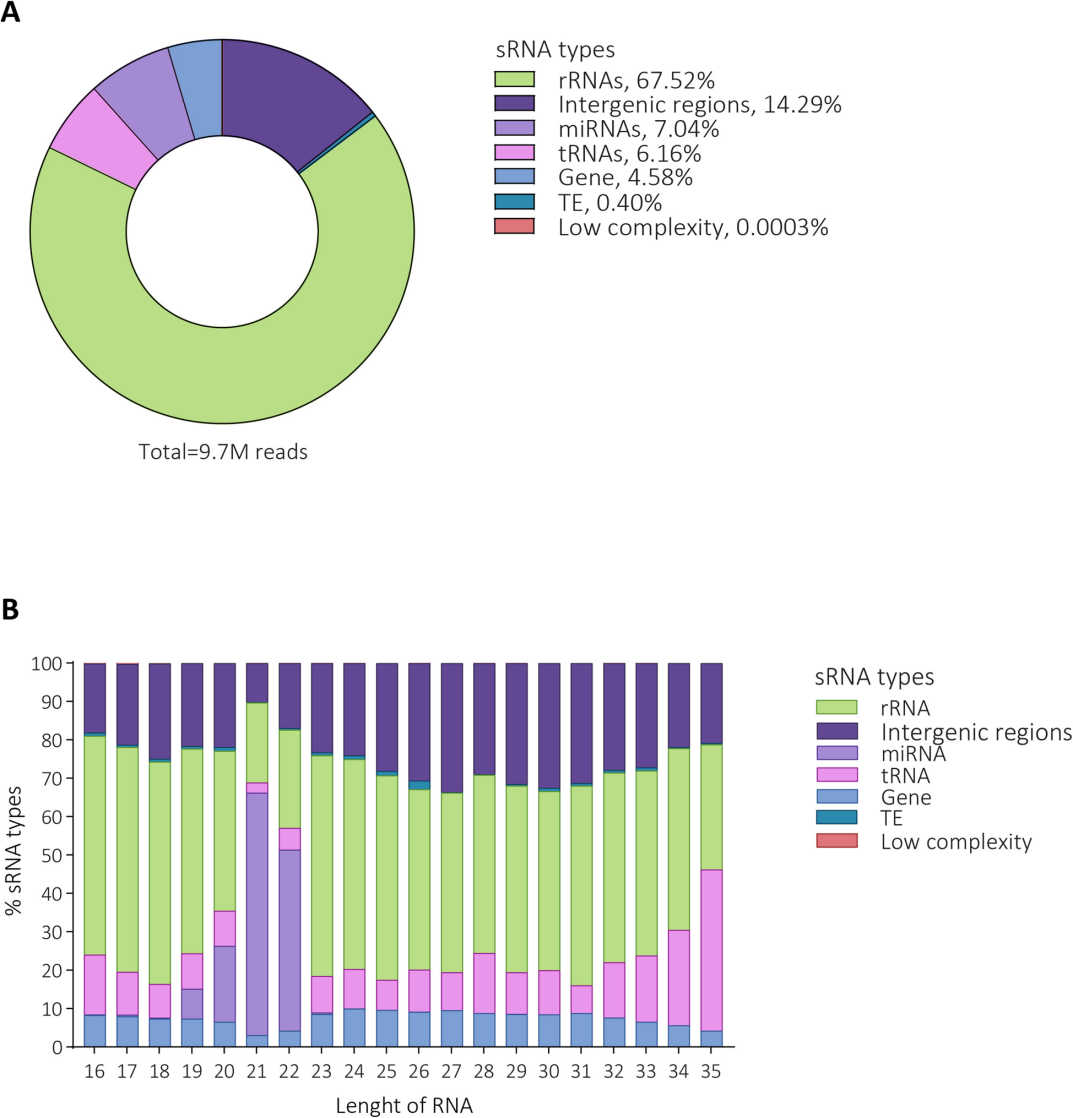
Abundance of the different sRNAs identified, expressed as percentage of reads, with respect to the total number of reads (**A**) or divided by sequence length (**B**)

Since a total of 520,297 unique sequences between 16 and 35 nucleotides long were identified, we further characterised the vesicular cargo of other classes of sRNA. First, we evaluated their 5’ base and sequence length (Supplementary Table S2). Overall, the 5’ nt base of sRNAs was AU rich. A 5’ adenine or uracil was present for 161,016 (30.9%) and 158,170 (30.4%) sequences, compared with 131,963 (25.4%) and 69,148 (13.3%) 5’ guanine and cytosines, respectively. Unique sRNA sequences of 18 nt in length were the most common and accounted for 41,469 unique sequences (8.0%). However, when considering the expression levels, shorter sequences were more abundant than longer sequences; 16 nt sRNA were the most highly expressed sRNA length comprising 11.82% of reads, and 34 nt sRNA were the lowest expressed comprising 1.62% of reads. The majority of expressed sRNA (67.5%) originated from rRNAs, followed by intergenic regions (14%), miRNAs (7%) and tRNAs (6%). The sequences of protein-coding genes constituted 4.5% of the total expressed sRNA reads (Supplementary Tables S2 and Fig. 3).

We used target complementarity to predict the targets of putative small-interfering RNAs (siRNAs), that is, sRNA sequences excluding miRNAs and low complexity reads. We identified 2498 human gene IDs with exact sequence complementarity to 1375 *S. stercoralis* iL3-EV putative siRNA sequences. The predicted human target genes were enriched for GO terms associated with neuronal development and function, histone modification, transcription and vesicular structures (Supplementary Table S2).

## Discussion

In this study we present, for the first time, experimental evidence on the release of EVs from *S. stercoralis* iL3s and provide the first characterisation of their smallRNA cargo by NGS. Of note, we investigated iL3s directly isolated from a clinical sample obtained from an infected patient, rather than from laboratory-maintained strains. Since the release of EVs by *S .stercoralis* has not been explored before, we first evaluated whether *S. stercoralis* releases EV sub-populations with different size ranges – distinguishable by differential ultracentrifugation. Our results, although requiring confirmation with orthogonal enrichment methods, indicate that in the first 48 h of in vitro maintenance at 26 °C, *S. stercoralis* iL3s release relatively small EVs with a mode diameter below 120 nm, which, consequently, are efficiently enriched at high centrifugation speed. Using NGS, we showed that EVs derived from *S. stercoralis* iL3 contain parasite-derived miRNAs and other sRNAs. Interestingly, EV molecular cargo only partly overlapped with *S. stercoralis* miRNome (70.6%) [23], suggesting that some might be preferentially packaged into EVs for extracellular release to interact with the external environment.

The growing importance of studies on helminth-derived EVs was highlighted recently by published technical recommendations [13]. Indeed, the ability of helminth parasites to survive and persist in the mammalian host is strictly associated with their capacity of modulating the host immune response [42, 43]. It is becoming evident that one of the weapons that helminths can exploit to carry out this task is the secretion of soluble biomolecules and EVs, collectively known as ESP [43, 44]. Our molecular characterisation suggests that iL3-EVs might be relevant for the interaction with the host in the early stages of infection.

As with other helminth-derived EVs, vesicles released from *S. stercoralis* iL3s contained mature miRNAs and other sRNAs. sRNAs are important regulators of gene expression at both the transcriptional and post-transcriptional levels [45, 46]. They have been reported to play an important role in both the interaction between parasitic helminths and their hosts [20, 47, 48], as well as in the regulation of larval development [49, 50]. Of note, in our dataset miRNAs represented only a small portion of the total expressed sRNA reads, whilst most sequences originated from rRNAs and intergenic regions. The predominance of rRNA- and the presence of tRNA-derived fragments have been described in EVs from other parasitic helminths and eukaryotic species, indicating that these RNA classes frequently constitute a major component of vesicular RNA cargo [48, 51], although the proportion of sRNA classes detected may be affected by EV enrichment strategies [48]. Nonetheless, the delivery of sRNAs to host cells has also been proposed as a key mechanism for cross-kingdom gene expression regulation [52] and for the modulation of host immunity to favour infection.

Although miRNAs were not the predominant sRNA species, our data confirmed that *S. stercoralis* EVs carry mature miRNA, in agreement with previous observation from other helminth-derived EVs [12]. In line with reports on *Heligmosomoides bakeri* and *Trichuris muris* [48], all predicted miRNA precursors from *S. stercoralis* iL3-EVs were located in intergenic regions, suggesting that they are likely transcribed from independent transcriptional units. This genomic organisation is also consistent with that described in *C. elegans*, where most miRNAs are intergenic and transcribed independently, whilst only a smaller fraction is intronic and co-expressed with their host genes [53, 54]. Several *S. stercoralis*-EV miRNAs were conserved across other nematode species, particularly *S. ratti* and *S. papillosus*, whilst others appeared *S. stercoralis*-specific. The identification of EV-specific miRNAs, such as sts-miR-175-5p, raises the possibility of selective sorting of sRNAs into vesicles; however, further studies are required to determine whether this represents an active regulatory mechanism or a stage-specific feature.

Interestingly, amongst the 319 loci from which precursor sequences originate, we identified two distinct genomic clusters. Although their proximity to protein-coding genes does not imply direct regulation, clustered organisation may facilitate co-transcription and coordinated expression, a phenomenon already observed in nematodes [55]. The bioinformatics prediction of human targets for *S. stercoralis* EV-miRNAs provided additional insights into their possible functional significance. Two miRNAs (sts-miR-172-5p and sts-miR-175-5p) were predicted to target seven human genes (*LGR6, DZIP3, PACSIN1, TRA2A, EPHB6, SHC3 and EMD*), whose expression profiles suggest distinct roles at the epithelial, immune and neuronal levels. *LGR6* and *TRA2A*, predominantly expressed in epithelial and gastrointestinal tissues, may be relevant to parasite entry and colonisation; whilst *EPHB6* – a regulator of epithelial adhesion and immune signalling – could contribute to immune modulation at mucosal barriers. In contrast, *PACSIN1* and *SHC3*, expressed mainly in neuronal tissues, and *DZIP3* and *EMD*, which have broader expression profiles, may be linked to systemic or extra-intestinal manifestations of infection. Comparative analysis indicated that sts-miR-172-5p shared sequence homology with *S. ratti* str-miR-34a-5p orthologue. sts-miR-172-5p, already identified in iL3 miRNome [23], belongs to the miR-34 family, which shows extensive evolutionary conservation across invertebrates and vertebrates. Although *C. elegans* encodes a divergent miR-34 sequence, homologous variants have been identified in several parasitic nematodes, including *S. ratti, Haemonchus contortus, Ascaris suum* and *Brugia malayi* [49, 50, 56]. The other miRNA predicted to target human genes, sts-miR-175-5p, was specific to *S. stercoralis* iL3-EVs, since it is shared neither with other nematodes nor with *S. stercoralis* iL3 miRNome. The targeting of human genes suggests a potential role for EV-derived *S. stercoralis* miRNAs in modulating host cellular processes and warrants further functional investigations to better understand the potential association with the mechanisms of host–parasite interaction and the pathogenesis of human strongyloidiasis. Consistent with miRNAs, other sRNAs were also predicted to target a wide range of human genes associated with regulation of gene expression and modulation of the immune system or neuronal functions. This large number of predicted targets is expected, as short sRNAs can match multiple transcripts with high complementarity by chance. Therefore, interpretation at the level of individual target genes should be made with caution. In this context, GO enrichment analysis was used to identify functional categories amongst the predicted targets. Whilst the enriched GO categories highlighted potential involvement in gene regulation, immune modulation and neuronal functions, results should be interpreted as exploratory and need further investigation.

Our study presents some limitations that should be mentioned. First, extending analyses to include EVs derived from multiple *S. stercoralis* clinical isolates will enable a more comprehensive characterisation of their biological variability and molecular cargo. Nonetheless, examining a clinical isolate rather than the commonly used laboratory strain PV001 strengthens the relevance of our findings. Second, the EVs characterised in our study have been harvested after larval in vitro maintenance. It has been reported that the release of EVs by helminths might change over time when worms are maintained in vitro [57], due to the lack of pressure coming from host factors. The effect of different in vitro conditions of *S. stercoralis* on the release of EV and their molecular composition should be explored more in depth in the future. An important additional consideration when studying helminth-derived EVs is the complexity of their life cycles, which encompass multiple distinct stages. Future investigations should include EVs from both parasitic and free-living *S. stercoralis* worms, although obtaining certain life stages from clinical isolates may prove particularly challenging.

Considering technical aspects, it is well recognised that the choice of the enrichment method may affect the sub-types of enriched EVs. Here we employed differential ultracentrifugation for the morphological characterisation and the kit for smallRNA profiling. More in-depth investigations, possibly employing orthogonal EV enrichment methods, should be performed to confirm our preliminary results and provide a more exhaustive description of *S. stercoralis*-derived EVs. Particularly, more extensive EV visualisation using cryo-TEM will help to more accurately determine their size distribution, the actual overlap between preparations at different UC speeds and the agreement with NTA measurements. Lastly, investigations should be extended to the characterisation of EVs protein cargo, to evaluate more in depth the potential functional role of parasite-derived EVs in the host–pathogen interaction during the first phases of infection.

## Conclusions

Here we provide the first experimental evidence that infective larvae of *S. stercoralis* isolated from an infected patient release EVs in the external environment. Our characterisation of their molecular cargo by next generation sequencing revealed that these EVs carry sRNAs with potential roles in host invasion and immuno-modulation, only partly overlapping with larval miRNome [23]. Although preliminary, our results pave the way for functional investigations into the role of iL3-derived EVs in host–pathogen cross-talk as well as for a more comprehensive evaluation of their potential clinical utility for the control of human strongyloidiasis.

## Supplementary Information

Additional file 1 (XLSX 61 KB)

Additional file 2 (XLSX 9574 KB)

## Data Availability

The data supporting the findings of this study are included within the article as supplementary information. NGS raw data have been deposited in the European Nucleotide Archive (ENA) under the study accession number PRJEB112850 and available at https://www.ebi.ac.uk/ena/browser/view/PRJEB112850.

## Abbreviations

iL3: Infective larva
ESP: Excretory/secretory product
EV: Extracellular vesicle
sRNA: SmallRNA
miRNA: MicroRNA
PBS: Phosphate buffered saline
UC: Ultracentrifugation
TEM: Transmission electron microscopy
NTA: Nanoparticle tracking analysis
GO: Gene Ontology
siRNA: Small-interfering RNA
